# What do parents think about the quality and safety of care provided by hospitals to children and young people with an intellectual disability? A qualitative study using thematic analysis

**DOI:** 10.1111/hex.13925

**Published:** 2023-11-28

**Authors:** Natalie Ong, Abbie Lucien, Janet Long, Janelle Weise, Annette Burgess, Merrilyn Walton

**Affiliations:** ^1^ School of Public Health University of Sydney Camperdown New South Wales Australia; ^2^ Child Development Unit Children's Hospital at Westmead, Sydney Children's Hospitals Network Westmead New South Wales Australia; ^3^ UNSW Medicine University of New South Wales Kensington New South Wales Australia; ^4^ Australian Institute of Health Innovation, Faculty of Medicine and Health Sciences Macquarie University Macquarie Park New South Wales Australia; ^5^ Department of Developmental Disability Neuropsychiatry (3DN), UNSW Medicine University of New South Wales Randwick New South Wales Australia; ^6^ Medical Education, Education Office, Sydney Medical School University of Sydney Camperdown New South Wales Australia

**Keywords:** children, hospital, intellectual disability, parent perspective, patient safety, young people

## Abstract

**Objectives:**

Children with intellectual disability experience patient safety issues resulting in poor care experiences and health outcomes. This study sought to identify patient safety issues that pertain to children aged 0–16 years with intellectual disability admitted to two tertiary state‐wide children's hospitals and a children's palliative care centre; to describe and understand these factors to modify the Australian Patient Safety Education Framework to meet the particular needs for children and young people with intellectual disability.

**Design, Setting and Participants:**

Parents of children with intellectual disability from two paediatric hospitals and a palliative care unit participated in semi‐structured interviews to elicit their experiences of their child's care in the context of patient safety. Thirteen interviews were conducted with parents from various backgrounds with children with intellectual, developmental and medical diagnoses.

**Results:**

Eight themes about safety in hospital care for children and young people with intellectual disability emerged from thematic analyses: *Safety is not only being safe but feeling safe; Negative dismissive attitudes compromise safety, quality and care experience; Parental roles as safety advocates involve being heard, included and empowered; Need for purposeful and planned communication and care coordination to build trust and improve care; Systems, processes and environments require adjustments to prevent patient safety events; Inequity in care due to lack of resources and skills, Need for training in disability‐specific safety and quality issues and Core staff attributes: Kindness, Patience, Flexibility and Responsiveness*. Parents highlighted the dilemma of being dismissed when raising concerns with staff and being required to provide care with little support. Parents also reported a lack of comprehensive care coordination services. They noted limitations within the healthcare system in accommodating reasonable adjustments for a family and child‐centred context.

**Conclusions:**

The development of an adapted Patient Safety Education Framework for children with intellectual disability should consider ways for staff to transform attitudes and reduce bias which leads to adaptations for safer and better care. In addition, issues that apply to quality and safety for these children can be generalised to all children in the hospital.

**Patient and Public Contribution:**

Parent advocates in the project advisory team were shown the questions to determine their appropriateness for the interviews.

## 1 BACKGROUND

Optimising patient safety involves addressing active and inherent risks within the complex interactions of the healthcare system; be it from an organisation or clinician perspective or a combination of both.^1^ Organisational factors are considered the ‘blunt end’, while clinicians are considered the ‘sharp end’ of producing errors in healthcare.^2^, ^3^

In the paediatric setting, the children most vulnerable to health care errors include those with intellectual disabilities. Intellectual disability is a term used when an adult, child or young person has certain limitations in cognitive functioning and skills, including conceptual, social and practical skills, such as language, social and self‐care skills and can occur any time before a person turns 22.^4^ It is now well established that people with intellectual disabilities suffer from poor health care outcomes arising from challenges in accessing and receiving safe and quality care.^5^, ^6^, ^7^, ^8^ There is growing evidence that health care staff themselves contribute to poor quality of care and adverse events.^9^, ^10^, ^11^ Clinician factors associated with unsafe care include poor communication and a lack of staff–parent interactions and partnerships.^10^ This is thought to stem from poor staff attitudes and biases towards parents and children with an intellectual disability.^12^ Parents often feel unheard, dismissed or excluded from decision making about their child.^13^ The child is frequently assumed to have little or no ability to understand or communicate, which can lead to them being unacknowledged or excluded.^14^, ^15^ Literature reports staff feeling uncertain, anxious and unskilled when it comes to caring for children with intellectual disability.^16^ However, in keeping with the social model of disability where inherently people, environments and systems are barriers to access for services and equal opportunity for children and adults with disability, there is a call to address this inequity in authentic and timely ways.^17^ Staff need to discard negative stereotypes and collaborate with parents, who possess tacit knowledge useful to clinical care.^18^

Raising staff awareness regarding the needs of these children and improving competency in patient safety is required to minimise unsafe care and adverse events.^10^, ^19^, ^20^ However, there is a paucity of literature reporting on parent and staff experience to inform competency framework development.

In 2004, The Australian Council for Safety and Quality in Health Care recognised the lack of a comprehensive education framework for patient safety was a barrier to achieving a competent and safe health workforce. In 2006, the Australian National Patient Safety Education Framework (APSEF) was developed, outlining specific competencies with regard to the delivery of safe and quality healthcare for the health workforce at the individual, service and organisational level. It was adopted by the World Health Organization which also produced a curriculum of competencies and recommended learning activities.^21^ While the APSEF had developed competencies for medications and surgery, no specific competencies associated with caring for children with intellectual disability were undertaken. This has been recognised by the authors, one of whom developed the original Patient Safety Education Framework. In addition, the membership of the project advisory group has representation from the Australian Commission for Safety and Quality in Health Care to inform the directions of the project. To expand the APSEF, we took a three staged approach and drew on multiple sources of data. First, we drew data from the literature by undertaking a systematic review of staff perspectives of safety issues for children with intellectual disability in the hospital.^10^ Second, we drew data from key stakeholders by conducting interviews with staff (Phase 2a) and parents (Phase 2b) of children with intellectual disability, structured around the existing domains of the Framework. In the final step, experts in the field will participate in a Delphi process to validate the themes and competencies that will constitute the new Patient Safety Education Framework for children and young persons with intellectual disability in the hospital setting.

This paper reports on the second phase of the study (Phase 2b), that is to elicit the experiences of parents and their understanding of safety for their children and identify themes and codes mapped to the national framework. This tranche of work identifies gaps in the current education framework and guides future programmes and education development for staff working with these children and their parents.

## 2 AIMS

### 2.1 The aim of this study is twofold

1. To identify patient safety issues that pertain to children with disabilities.
2. To describe these issues to inform adaptations to the APSEF domains and subdomains for this population.

### 2.2 Our specific research questions were

1. What were the patient safety‐related issues experienced by the parents regarding their child's hospital care?
2. How do these themes help us understand missing competencies and understandings needed for the adapted APSEF?

## 3 METHODS

Participants were parents of children and young people with intellectual disability who attend one of two tertiary state‐wide children's hospitals and a children's palliative care centre. Expressions of interest through invitation flyers circulated through parent networks (10) and patient and family engagement teams (3). A snowballing approach was undertaken where study participants made recommendations for other parents to be included in the interviews. Inclusion criteria included being a parent of a child who was a patient of the tertiary children's hospital, having had an emergency presentation, admission, clinic attendance or procedure in the last 12 months. Exclusion criteria included parents whose child did not have an intellectual disability or no hospital encounter in the last 12 months. Recruitment of parents and staff occurred simultaneously in April 2021 to May 2022.

An interview guide was developed after consultation with the primary author of the APSEF^1^ and the research team. In the initial part of the interview, open‐ended questions were used followed by more focused questions based on the framework. This opened up opportunities for new or additional themes that may not arise by adhering too closely to the framework. Following which questions were then derived from the domains of the Framework to obtain insights pertaining to the domain in the context of a child with intellectual disability in hospital. For example, the question: ‘Can you tell me of an experience of good/bad communication in a hospital setting?’ related to Domain 1 of the APSEF, communicating effectively, and sought to understand issues for this cohort of patients. An initial question, ‘What does the word safety mean to you?’ was asked to elicit aspects of safety that may be relevant to parents but not considered by staff or the APSEF.

Due to the COVID/lockdown restrictions, recruitment was delayed. Semi‐structured interviews were conducted by the primary author (N. O.), a developmental paediatrician with over 20 years in clinical practice. The duration of the interviews ranged from 35 to 77 min, (35:46; 1:04:46; 49:08; 51:28; 63:41; 41:48; 49:15; 1:00:14; 36:34; 77:46; 1:02:49; 48:34; 51:37). The interviews were audio recorded and transcribed. All identifying information was removed at transcription. The interviews ceased upon data saturation as agreed with the primary author and the research team. The interview guide is presented in Table 1.

**Table 1:** The Australian Patient Safety Education Framework.^21^

**National Patient Safety Education Framework**
1. *Communicating effectively*
1.1 Involving patients and carers as partners in health care
1.2 Communicating risk
1.3 Communicating honestly with patients after an adverse event (open disclosure)
1.4 Obtaining consent
1.5 Being culturally respectful and knowledgeable
2. *Identifying, preventing and managing adverse events and near misses*.
2.1 Recognising, reporting and managing adverse events and near misses
2.2 Managing risk
2.3 Understanding health care adverse events and near misses
2.4 Managing complaints
3. *Using evidence and information*
3.1 Employing best available evidence‐based practice
3.2 Using information technology to enhance safety
4. *Working safely*
4.1 Being a team player and showing leadership
4.2 Understanding human factors
4.3 Understanding complex organisations
4.4 Providing continuity of care
4.5 Managing fatigue and stress
5. *Being ethical*
5.1 Maintaining fitness to work or practice
5.2 Professional and ethical behaviour
6. *Continuing learning*
6.1 Being a workplace learner
6.2 Being a workplace teacher
7. *Specific issues*
7.1 Preventing wrong site, wrong procedure and wrong patient treatment
7.2 Medicating safely

The data was analysed using Braun and Clarke's method of thematic analysis.^22^ The initial data familiarisation through reading and data immersion of the transcripts was done to generate deeper insights. Then codes were generated for as many topics as possible in the second round of reading. These codes were contextualised and classified into categories. The categories were further aggregated to generate themes. The themes were further defined and named with corresponding quotes as illustrative examples in Table 2.

**Table 2:** Interview guide.

Introduction
Parent age, occupation, child age and diagnosis
What does the word safety mean to you? What does it mean to have safe care in hospital? Can you tell us your experience of what went well? What didn't go well in hospital for your child?
(Optional) I would like to encourage you to think about (1) What it was that staff did in (or not) those instances? (2) Was the issue related to a practice, procedure, or things in the environment? (3) What were the things that led to the issue? Could it have been prevented?
When thinking about patient safety standards there are seven domains—these care standards of care to ensure that your child receives good and safe care. We want to promote good practice and strive for positive and good experiences, not only to avoid mistakes or near misses.
The first relates to communicating effectively to you and your child. (APSEF Domain 1: Communicating effectively)
Can you tell me of an experience of good/bad communication in a hospital setting?
The second relates to identifying, preventing, and managing when something goes wrong or a ‘near miss’ has occurred. (APSEF Domain 2: Identifying, preventing, and managing adverse events and near misses)
Can you tell me of an experience where something went well/went wrong or almost went wrong?
How was it communicated to you? If there was a better way around it, how would you and your child like to be told about it? What sort of things would you expect the health worker to be able to do? How can things be done differently the next time or for others?
The third relates to using evidence and information in hospital practice. (APSEF Domain 3: Using evidence and information)
What would you like health workers to know in regard to caring for a child or young person with intellectual disability? What do you think would be the best way for health workers to learn how to care for a child with intellectual disability safely in hospital? Are there any technological advancements that can be used to help?
The fourth relates to safe work practices. (APSEF Domain 4: Working Safely)
What are the types of things that would make for safe work practice that you may have noticed? For example, this could relate to the way the team worked together, or certain work practices that was good to prevent mistakes from happening? How do you think the team managed continuity of care? How do you think they managed stress and fatigue? Can you think of some examples where things were done well? Or times when things we not done well which made you feel uncomfortable and anxious about your child's safety?
The fifth relates to core attributes of staff caring for a child or young person with intellectual disability. (APSEF Domain 5: Being ethical)
How would you like staff to treat you and your child and what skills abilities and attitudes should they have? Do you think staff behaved in a professional and ethical way?
The sixth relates to continuing learning. (APSEF Domain 6: Continuing Learning)
As a parent of a child with intellectual disability, there is a lot we health professionals can learn from you. How do you think the health workforce can learn from your experience of your child? How do you think they can be effective teachers of how to care for your child?
The seventh relates to specific topics in patient safety I would like to explore. (APSEF Domain 7: Specific Topics). The first relates to medication safety. Would any one like to talk about relevant experiences? The next relates to prevention of operating on the wrong site, having the wrong procedure or wrong treatment. If your child experienced any of the above, were there any ways that these could have been prevented? Are there any other areas or topics that need to be included?

Abbreviation: APSEF, Australian National Patient Safety Education Framework.

**Figure 1:**
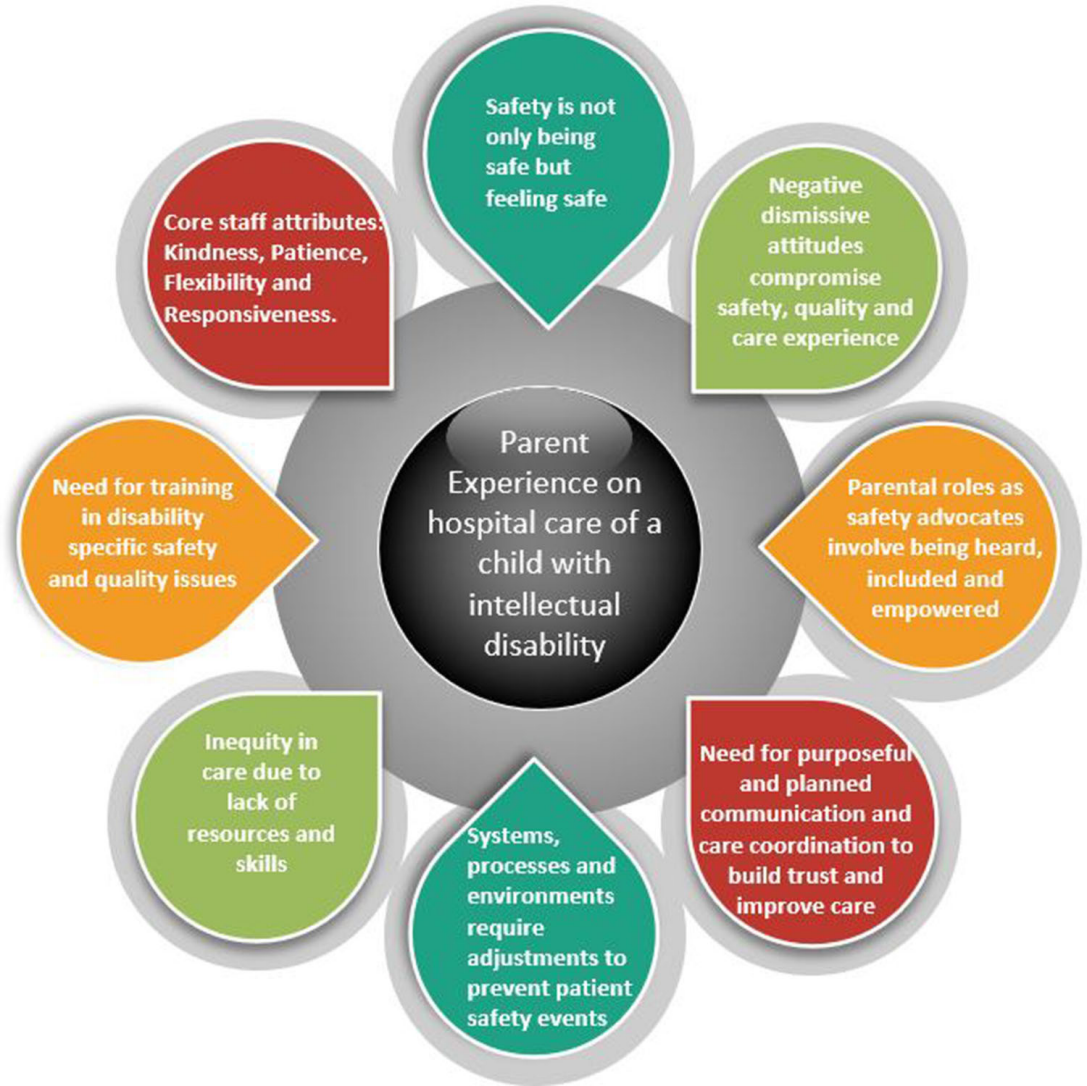
Conceptual framework for parent perspective of patient safety themes for children and young people with intellectual disability.

The lead researcher (N. O.) coded the transcripts and developed the initial code set. Another researcher (A. L.), a psychologist who works in a Child and Adolescent Mental Health Unit, conducted independent coding of the same transcripts using and refining the code set. Similarities and discrepancies were discussed with the research team and resolved through discussions and meetings (Figure 1).

### 3.1 Ethics approval

Ethics approval was obtained through the Sydney Children's Hospital Network Hospital Research Ethics Committee. Protocol No: 2020_ETH02240.

## 4 RESULTS

Of the 13 parents interviewed, three were male and 10 female. The mean age was 40.9 years, with the age range between 32 and 52 years. Family demographics were not reported to preserve confidentiality. Their children's ages ranged from 5 to 14 years, all had a diagnosis of intellectual disability as well as other syndromes, co‐occurring autism, and medical conditions.

Eight key themes and 11 qualitative categories were derived from the framework analysis from interviews of parents of children and young people with intellectual disability which include: *Safety is not only being safe but feeling safe; Negative dismissive attitudes compromise safety, quality and care experience; Parental roles as safety advocates involve being heard, included and empowered; Need for purposeful and planned communication and care coordination to build trust and improve care; Systems, processes and environments require adjustments to prevent patient safety events; Inequity in care due to lack of resources and skills, Need for training in disability specific safety and quality issues and Core staff attributes: Kindness, Patience, Flexibility and Responsiveness*.

### 4.1 Theme 1: Safety is not only being safe but feeling safe

When parents were asked what patient safety meant to them in the context of their child with an intellectual disability in the hospital, most parents stated that safety was the elimination of physical, emotional and psychological harm and the avoidance of distress, hurt or harm. One parent commented that patient safety also extends to ‘what people can do to make the experience better’ (P9) *and allowing parents/patients to be* ‘well‐informed, able to make clear choices and knowing the full consequences of those choices and decisions and having time … to consider those decisions’ (P6). Having a timely response to intervention or onset of symptoms and having effective and ongoing management from a team that is coordinated, cohesive and collaborative was also thought essential. A parent with an adolescent with intellectual disability and challenging behaviours added ‘I want him to get there without us struggling and fighting with him’ (P8).

Illustrative quotes and subthemes are shown in Table 3.

**Table 3:** Safety is not only being safe but feeling safe.

Aspects of safety	*There is two aspects to safety. There's the physical safety and then there's the emotional safety*. (P1)
Taking precautions to reduce harm and distress	*Making sure that every precaution is taken to keep a child alive and safe, free from harm and not to cause any further distress or hurt or harm …. that also extends to what people can do to make the experience better, easier, more manageable, et cetera*. (P9)
Providing adequate information for parents to make informed choices	*Is well‐informed, being able to make clear choices and knowing the full consequences of those choices and decisions and having time, if possible, if the situation allows, to consider those decisions*. (P6)
Timely response and effective team coordination and collaboration	*Having a timely response to intervention or timely response to onset of symptoms. Having effective and ongoing management from a team that is coordinated and cohesive and collaborates together*. (P6)
Prevention of physical, emotional and psychological harm	*The first thing is the physical safety and prevention of harm physically, or emotionally, or mentally. In terms of the children, obviously, it's a bit more [hands on] action. So that's generally, and in the [hospital] it's kind of the same. It's really just avoiding further harm. [If you're in] hospital there's a reason [you're there] and it's about preventing any additional issues*. (P7)

*Note*: Key illustrative quotes from interviews.

### 4.2 **Theme 2: Negative dismissive attitudes compromise safety, quality and care experience** (of relevance to Domains 1: Communicating Effectively, 2. Identifying, preventing and managing adverse events and near misses and 4. Working safely)

Parents with intimate knowledge about their child with intellectual disability, felt their knowledge was underutilised. At times, they felt staff dismissed and ignored their concerns which had an impact on the quality and safety of care received. A few parents described how their feedback to staff was ignored, stating that this led to an adverse event for their child with intellectual disability. Parents observed that staff failed to recognise that not all children with intellectual disability are the same and that such assumptions inhibited staff seeking premorbid information about the child. When a child presented with subtle behavioural changes, parents found themselves needing to convince staff not to be sceptical, noting that this reluctance to listen to them resulted in adverse events.

Parents said that communication tools and strategies could help staff engage with their child with an intellectual disability more effectively. They suggested simplified language, basic signing, social stories or visuals should be part of standard care provision when communicating tests or procedures to their child. Parents reported occasions when asked by staff how their child communicates led to a more positive experience due to the reassurance that the staff would check in directly with their child. Parents report that their child with intellectual disability also need appropriate activities to relieve boredom, alleviate anxiety with activities familiar to them. When staff gave time and attention and were familiar with the child‐conducted procedures, or play therapists were engaged, the care experience improved. Although the inclusion of play therapists in the acute settings such as an emergency department can help alleviate anxiety associated with procedures,^21^ this was not always possible in situations when staff were stressed and busy managing clinical duties.

Parents of children with an intellectual disability wanted to be asked on how to manage their child's behaviours that challenge as the strategies are often highly individualised. However, they report that this does not often occur.

Parents understand that other parents with children with intellectual disability may have not respected staff leading to staff having negative attitudes as a result. They feel the junior nursing staff are more willing to ‘go the distance’ compared to more senior nursing staff.

Illustrative quotes and subthemes are shown in Table 4.

**Table 4:** Negative dismissive attitudes compromise safety, quality and care experience.

Recognising parents as experts of their child	*He was for six days admitted as a psych patient where we were told he had bipolar disorder and sedated and. I think it was day six, I insisted that he get examined by someone because there was something physical going on … the behaviour, just incremented until his face ballooned and he had three septic teeth*. (P12)
Customisation not assumption	*[if]mum is saying this is different for [my] child I think is a really big alert and it has to be given a little bit more credence … and so we're very hypervigilant with our kids, probably often ‐ I don't know, looking, seeing the subtle changes*. (P7)
Need to use communication tools and strategies	*A little bit of understanding of alternate communication, like key word sign or PECs, using the picture symbols to communicate with children or adults would be perfect to have on a lanyard*. (P2)
Engagement and familiarity leads to improved experiences	*…because if the child is comfortable and not agitated, not crying and all that, the procedure runs smoother and safety is then taken on board and it makes things a lot easier. So, they brought a play therapist in, they went through what was going to happen, they gave A a toy to play with and they blew bubbles …. Something so simple but it made a big difference*. (P9)
Providing activities to prevent boredom and anxiety	*Again, just anything that distracts your child for a bit … you're there but you're not needing to come up [with things to entertain constantly]*. (P3)
Anchoring bias	*So then that gives you an indicator, okay, is there something more to this and not just, oh, this kid is just a whinger or this kid just likes the attention. That comment, that attention seeking comment, I hear so many times it just gets to me*. (P2)
Premature closure	*They assume that everyone is the same, and the assumption is not a good thing…. You don't know where this child is. They're only eight months or a year…[level in ability despite chronologically older]. So that miscommunication and that assumption can lead someone astray…* (P2)
Implicit bias	*Why is there that bottom line assumption that defaulting people with ‐ like J to psychiatry rather than looking at the organic causes that might be there*. (P8)
Understand previous negative experiences for staff influence attitudes	*I think this eagerness to please the parent and work with the parent fades and I can understand that because some people are just not nice. I've seen it in the hospital. Some parents are not nice … So I think when they encounter assertive parents … They get a bit defensive. That's what I've noticed. Whereas the younger ones they're just starting in the field and they haven't gone through all the trauma…* (P1)
Senior versus junior attitudes	*Many nurses understand that perfectly and they work with you really well. But sometimes you do encounter – and I don't know why this is. Sometimes – it's usually – I think the more senior they get, the more impatient they get*. (P1)

*Note*: Key illustrative quotes from interviews.

### 4.3 **Theme 3: Parental roles as safety advocates involve being heard, included and empowered** (of relevance to Domain 1: Communicating Effectively, 2. Identifying, preventing and managing adverse events and near misses and 4. Working safely)

On one hand, parents of children with an intellectual disability felt staff relied on them to provide care, for example, needing to check medication doses and that the correct procedure(s) were performed, as well as identifying lapses and mistakes. However, when such issues were identified and raised by these parents, they felt judged, disbelieved or dismissed when trying to inform staff. One parent reported that complications arose when their concerns were dismissed by staff.

Parents felt that their presence was to mainly ensure their child with an intellectual disability felt secure in the hospital. However, some parents observed staff often failed to provide safe care amid busy clinical environments, noting a lack of continuity of care if they were absent, thus necessitating their continual presence to ensure that aspects of care were not overlooked.

Parents said at times they felt overwhelmed and stressed and did not receive support for themselves given the demands on them to always be present. They felt the expectation to provide 24/7 care while their child with an intellectual disability was in the hospital unrealistic, noting they had to juggle other responsibilities, such as caring for other family members.

Parents reported that information and progress updates or consent alleviates anxiety and helps manage expectations. They identified ‘handover’ as time when they could assist staff to check information and participate in clinical decision making. Parents reported feeling disenfranchised when not receiving regular updates about their child's care and treatment, fearing that clinical decisions were being made without considering parental concerns or their child's best interests. According to the parents, information about other relevant hospital services was not made available, leading to a reliance on their own networks for information.

Insensitive comments made by staff was another issue raised by parents of children with intellectual disability. They were reluctant, however, to confront staff regarding this fearing repercussions that might impact their child's quality of care. Parents do not always feel empowered to escalate their concerns and feel some frustration in trying to convince staff of additional tests or investigations for their child with intellectual disability.

Illustrative quotes and subthemes are shown in Table 5.

**Table 5:** Parental roles as safety advocates involve being heard, included and empowered.

The paradox of parents as safety advocates	*If I hadn't have gone into bat for her and then an emergency did happen, who would be responsible and it could have been prevented just by them listening to me in the first place*. (P9)
Trust and role negotiation works both ways, keeping informed, listening, performing procedures	*Someone whom [I can] trust. Trust is the number one thing. Willing to listen when I'm concerned about something. Act when I request action about something*. (P1) *They do understand very well what it means to work as a partnership with the parent at such point that when I get into the hospital and once I'm in the ward and once I talk to the surgical teams, they talk to the nurses and they pretty much brief them just be guided by what she says. She will – I do the washouts, basically, and the surgeon doesn't need to be there. I just need a nurse to help me. So there's a trust system between the surgeons and us. Very well established for the last six years. So communication is very good*. (P1)
Keeping parents informed in a timely way alleviates stress and improves safety and quality of care	*I think a fluid communication line with the procedures that are happening with the child at that moment and leading up to it … I find myself apologising for asking so many questions because I don't know if anyone else does. So, for me the better equipped I am for the day…* (P2) *There's no point in me waiting six hours in the hospital for something that I can resolve … I just need to talk to the surgeon. Sometimes it's about how many washouts do I need to do or something. A very simple question*. (P1)
Communicating with sensitivity and honesty	*N was very little when she was born. At three months old I get told; have you been feeding her? She looks like a newborn*. (P2) *They're not being truthful about the beds or the way the beds are funded or anything like that … It was about budgets, and staffing, and everything else*. (P10)
Need to keep things positive	*So to me keeping a good relationship with the nurses is number one thing because I will be there many times so I don't want to get in trouble with them*. (P11)
Inclusion during handover	*… I think if you avoid the parent and do handover outside or not even look at the patient and shut the door and talk to the nurse during handover outside, like nurse to nurse, then things can get missed*. (P9)
Empowerment to escalate varies	*…so I was able to talk to the senior person there. When things don't go well, I know I can bring it – I can escalate it. I am aware of that. P11* *So over the year from then we came to find […autism] as she grew up ADHD, OCD, sensory processing disorder, feeding problems that we had never thought were an issue…But then to know that your child is actually silently aspirating was so dangerous. Then to convince specialists to carry out certain tests for her was a nightmare sometimes*. (P2)

*Note*: Key illustrative quotes from interviews.

### 4.4 **Theme 4: Need for purposeful and planned communication and care coordination to build trust and improve care** (of relevance to Domain 1: Communicating Effectively 2. Identifying, preventing, and managing adverse events and near misses 3. Using evidence and information and 4. Working safely)

Parents of children with intellectual disability reported that effective communication occurs when staff listen and are responsive to parental knowledge, skills, and concerns. This then results in a relationship of trust, one in which parents can assist in providing technical care that they already undertake at home. In turn, they felt this resulted in parents allowing staff to provide care needs and respond to their child's cues.

Parents recognised the importance of effective teams and emphasised the importance of effective and accurate transmission of clinical and care information of their child with intellectual disability through collaborative communication to reduce errors and adverse events. If mistakes are made in communication, a culture of parent inclusion would reduce the likelihood of errors perpetuating in handovers and clinical practice.

Many parents of children with intellectual disability reported key strategies, such as checking for information before admission, receiving support from staff familiar with child, having enough staff to provide supervision and care should be adopted. Parents said that if staff knew about their child before an admission, better planning for the right environment, equipment and appropriate staff allocation could be arranged. Parents of children with intellectual disability saw value in the use of the hospital passport (summary document outlining child's likes, dislikes, communication, behavioural triggers and care needs) and other resources of information exchange, but they were not utilised enough.

Parents of children with intellectual disability understand the need for staff to triage parent communications with the specialist postdischarge. However, parents report that due to inadequate time and institutional barriers, they are often excluded in these discussions which can lead to unnecessary admissions or even in obtaining a specialist report.

Parents of children with intellectual disability from rural remote areas report travelling long distances for appointments and access to health services may be more limited. Additional considerations on discharge are needed to ensure that they are discharged with adequate support.

Medication safety issues for children with intellectual disability related to lack of familiarity with psychotropics and side effects with incidents ranging from dosing errors, poor adherence to guidelines or poor checking processes.

Illustrative quotes and subthemes are shown in Table 6.

**Table 6:** Need for purposeful and planned communication and care coordination to build trust and improve care.

Importance of parent inclusion, team coordination and liaison, care coordination, team communication	*A plan where everyone's informed …. a group of professionals picking up the phone or having a group email and firing off a two line email saying hey, what does everyone think about this?* (P6) *Whereas when the nurse does handover at the bedside a nurse goes ‘mum, have I missed anything? Is there anything else that we can add on?’* (P9)
Checking information beforehand allows preparation, efficiencies and better care	*One of the nurses did say she already had looked up her condition and she has done a bit of research on her accord, which was beautiful to hear*. (P2) *I don't know. I just feel you've got a checklist, but those sorts of things should have happened before she got brought to the ward. That literally when she got to the ward she could just go to sleep, but instead we were all fluffing about trying to get everything in place. It's there. It's just carrying out the actions is the issue*. (P)
Using the hospital passport	*…but maybe an alert card. So, people who are new to the healthcare system or sort of novice workers might be alerted or triggered and just say, like a little health pass to say, this is my name, like an all about me, wallet sized all about me, identifier for the hospitals to say this is my hospital ID and all ‐ do you know what I mean?* (P5)
Coordination of multiple appointments is challenging	*I guess the main challenge for us has been because of the amount of people that they are looking after her is the coordination of appointments. I found Outpatients Department very difficult to liaise with. Sometimes letters, they don't arrive. Sometimes appointments are not made. Sometimes the waiting in the rooms ‐ and I know it's a public hospital but some of the times is just ridiculous*. (P6)
Patients from rural and regional areas	*… D was beyond any ENTs in a regional area, they couldn't care for him. So, we ended up coming back there, a lot – we've well beaten the path, life is a highway in the car. We spent many a time driving to Sydney, lots of time on the road and down there in the hospital*. (P10)
Medication safety issues still occurs	*In the mean time, he lost his hearing and we were lined up for a cochlear implant for both sides and it wasn't until [dad] sort of did a Medline search and found one guy in India had had a negative side effect to Olanzapine and had lost his hearing that we spoke to the ear specialist and he said, come off the Olanzapine and the hearing recovered to pre‐Olanzapine levels…* (P6)

*Note*: Key illustrative quotes from interviews.

### 4.5 **Theme 5: Systems, processes and environments require adjustments to prevent patient safety events** (of relevance to Domain 2. Identifying, preventing and managing adverse events and near misses 6. Continuing learning and 4. Working safely)

Parents of children with intellectual disability report inadequate medical or procedural information leads to increased risk of a failed or traumatic procedure, noting that electronic database systems should be linked across health facilities.

Currently, hospital IT systems are not shared with general practice IT systems and disability systems. This makes the transfer, updating and access of information across sectors challenging. For example, a general practitioner (GP) or a paediatrician may have started a new medication or conducted blood tests with results not available in the hospital system. Likewise, when there is a discharge summary, it has to be faxed to the GP or posted. Few practices have email facilities due to cyber security issues around confidentiality.

Hospitals are yet to develop systems capable of identifying children and young people with intellectual disability who may need access to fast‐tracking procedures and treatment adjustments. Such a mechanism would enable staff to contact families' beforehand, prepare for reasonable adjustments and fast track those who are anxious in busy waiting areas thus minimising risk of adverse events. Judicious booking systems, reducing long waits, working quickly, preparation with social stories and other opportunities to acclimatise were some of the suggestions found to be helpful.

Parents of children with intellectual disability spoke of environmental and equipment issues such as no child‐sized toilet seats, lack of unreachable door handles to prevent a child absconding. Environmental hazards such as open bins and treatment rooms must be closed and locked. Having alternatives to noisy, crowded waiting areas, open‐plan emergency cubicles and providing the option of taking the child for a walk to a quiet place would be helpful. Having the right equipment and easy access to toys, foods and drinks, signage if child is unable to feed or clean him/herself is also relevant.

Evidence of staff responding to adverse events was reported by one parent, which had resulted in staff working on a policy document to such prevent further events from recurring.

Illustrative quotes and subthemes are shown in Table 7.

**Table 7:** Systems, processes and environments require adjustments to prevent patient safety events.

Lack of linkage in accessing medical records is problematic	*I went, oh, what happened? The anaesthetist came out all very, very sorry. obviously had done kids as well but he couldn't access D's notes from [other hospital]*. (P10) *Now what I would love to see is an opt‐in service where you can have the specialist, the GPs and the allied health people all opt‐in to have access to what medication change or what [information] ‐ what's relevant to [my child]‐ and wouldn't that be great if it was contemporaneous. So I go to the paediatrician today or psychiatrist today with J. They change his [medications]. and [this] goes straight into each [electronic system]*. (P6)
Means to flag and fast track	*The waiting list for a scan under general anaesthetic is, as you know, quite long and sometimes up to 10 months. I get the reasons for that but I also think there must be at some point some consideration around fast tracking children or adults with disability who have ongoing health issues that are escalating and maybe trying to push that forward…* (P6)
Equipment and space matter	*So with the physical safety is ensuring that she's not going to fall off the bed, that she can keep her cannulas on and that she is not able to escape the room when she's in the ward and that she can sit safely on a toilet which is currently not designed for children. I don't understand that*. (P11)

*Note*: Key illustrative quotes from interviews.

### 4.6 **Theme 6: Inequity in care due to lack of resources and skills** (of relevance to Domain 2. Identifying, preventing, and managing adverse events and near misses 3. Using evidence and information and 4. Working safely and 5. Being Ethical)

Parents of children with intellectual disability said they had to access private services due to long wait times for clinic appointments and urgent and elective procedures. At times the public health service did not cater well for children with rare genetic conditions leaving parents to learn all about the condition and ‘inform’ the doctor what to look for and relevant comorbidities. Having to repeat information to health professionals who have limited time to provide comprehensive care was noted.

Delays in diagnosis and treatment frequently occurs as children with intellectual disability are often lost in the system. The failure in care could relate to a missed process or a reschedule because an exam or test could not be performed due to behaviours that challenge. New ways of managing and investigating patients where report on symptoms and physical examination is challenging is required.

Parents of children with intellectual disability feel that they need to manage this risk and find relief when they have access to a contact person or colleague in the hospital to assist.

Illustrative quotes and subthemes are shown in Table 8.

**Table 8:** Inequity in care due to lack of resources and skills.

Needing to access private care for timeliness	*So I find myself still to this day advocating and battling specialists to give us the direction or the right testing for her*. (P2)
Delays due to inability to progress appointments or investigations	*Yeah and following up, I think someone like actually sort of not only ordering a test but actually checking in and saying well okay, so we got nothing there. So handing over the baton. If they can't run with that baton, they hand it over and make sure someone else takes it on. It's like actually here's the baton and they're going to run with you and this is them and they'll see you in two weeks or…. more timely because invariably these guys and the data all shows this, these guys, they're delayed in their treatments. I think you must ‐ yeah, that ‐ delayed diagnosis because they're not verbal*. (P7)

*Note*: Key illustrative quotes from interviews.

### 4.7 **Theme 7: Need for training in disability‐specific safety and quality issues** (of relevance to Domain 3. Using evidence and information and 6. Continuing learning)

There were some reports of positive experiences in the form of families' satisfaction of high‐quality specialist care in managing their child with intellectual disabilities medical conditions. Some parents report noticing improvements in coordination of care for their child, and the way their child was treated and managed in the emergency department. Positive feedback was received on the experience of blood tests, feeling understood by staff, and reports of good communication, collaboration and child engagement were reported by a few parents. Parents of children with intellectual disability appreciated the presence of play therapists and clown doctors in distracting and alleviating anxiety for their child and would like to have more opportunities for such services.

For the most part, parents are aware that staff lack training and might feel overwhelmed when caring for their child with intellectual disability. While communication skills in managing patients with an intellectual disability are not taught to health professionals, parents appreciate efforts by staff to go the distance to engage their child.

Sometimes staff do not know how to manage a child's behaviour or how to prevent or de‐escalate it. Parents reported that due to the range of presentation that a child might exhibit that talking to the parents is a useful way to learn how to manage behaviours that challenge. Staff can learn from them or from other skilled staff, for example, pathology on blood taking, dental staff for alleviating anxiety, and so forth.

Parents acknowledged that staff could feel stressed and overwhelmed, balancing work in a busy ward, compounded by the care of a child with an intellectual disability and complex medical needs. This increases the risk of errors. Training in specific skills eg. tracheostomy care, skills in assessing the child's state, use of communication tools, behaviour strategies or other forms of reasonable adjustments, were thought to be needed.

Illustrative quotes and subthemes are shown in Table 9.

**Table 9:** Need for training in disability‐specific safety and quality issues.

Treating with dignity	*I guess, if they're not trained, it's probably going to be hard [laughs] and overwhelming. So, I said, please just be a bit more practical [unclear]. Just don't get overwhelmed with seeing, whenever you are seeing and just trying to adjust to the situation the best you can, I guess. Treat them with some dignity, I guess*. (P10)
Specialised skillset lacking	*For me, we're going on eight years and we've only managed to find two nurses that are adequately trained to care for A and provide me some sort of respite*. (P4) *These kids are people, not just to treat them like the notes, or the handover notes, or what the last nurse has said. To learn about the child as they go*. (P9)
Training junior staff by modelling appropriate skills, attitudes and behaviours	*Practice what you preach. Going through nursing school or doctor school or whatever, how long do you have to shadow someone? When you are training others make a little bit of extra effort*. (P4)

*Note*: Key illustrative quotes from interviews.

### 4.8 Theme 8: Core staff attributes: Kindness, Patience, Flexibility and Responsiveness (of relevance to Domain 4 Working Safely and 5. Being ethical)

Parents of children with intellectual disabilities report staff qualities such as kindness and empathy resulted in positive experiences. Such staff tended to give time and attention, were flexible, patient, good listeners and responsive to the needs of the child and their parents. These qualities helped to instill trust by parents. Staff who engaged with their child allowed them to feel more comfortable. Parents preferred staff who did not place judgement or make assumptions and were willing to ‘go the distance’.

Illustrative quotes and subthemes are shown in Table 10.

**Table 10:** Core staff attributes: Kindness, Patience, Flexibility and Responsiveness.

*Core attributes*	
Kindness and empathy	*Compassion, I guess, for the situation and the child. Caring and ‐ caring and kindness and understanding that things might take a bit longer or might need a little bit of extra help or situations don't always work out as expected with kids with intellectual disabilities but I think just compassion in general*. (P4) *I think the empathy was quite clear. It was I think what stood out for me…. there was a young emergency doctor who was amazing and there was ‐ like right from the beginning but also the nursing staff were really good*. (P6)
Give time and attention	*Yeah, your vibe. If you're rushed, if you're not in the mood, you haven't had a good day, kids will go off that, and I've seen it firsthand with N. But if you give them that attention that they need, if you've done a little bit of a background search onto these kids and understood their disability and ability these children will actually give back*. (P2)
Need to be flexible, patient, responsive and a good listener (five files, three files, two files)	*They need to be compassionate and I think patient and flexible, I guess, flexible in terms of their thinking and their action plans and things like that. … obviously good communicators*. (P12)
Know how to effectively engage the child to make them comfortable	*That ability to engage with a child, especially a child with a disability like my kids. Being able to engage them will disarm them quite a lot, and it makes the experience better*. (P7) *… for those that are perhaps nonmobile or non‐verbal it becomes more ‐ more emphasis is needed for the person who's doing the care to have a personality that's easily adaptable. To have the personality that can make jokes or be childlike*. (P9)
Trusted by parents	*So not to box kids, is not – and I think it's – in a manner it is unsafe to box a child as autistic because as you know and I know, not everyone – no autistic child is the same. They're all different. If D doesn't like something and some other autistic child does like it, should not presume that any child with an intellectual disability would like or not like something*. (P10)
Withhold judgement	*‐ that sense of being understanding, being open‐minded, not to allow oneself to be too overwhelmed or too emotional about the situation. You just have to set your own personal things aside and really look at the child for their situation and try to understand their situation to be helpful.…* (P8)
Willing to go the distance	*They have to do more than the minimum and they don't, 99 per cent don't do more than the minimum. I don't know how many times I've walked in and found Milky Bars [unclear] on D's lunch trolley. There's nobody here to help [lunch], it's still sitting there next to his bed…* (P10)

*Note*: Key illustrative quotes from interviews.

## 5 DISCUSSION

This study developed codes and themes from semi‐structured interviews with parents of children with intellectual disability from a range of backgrounds to highlight specific patient safety issues for their child with intellectual disability in the hospital. We sought to develop a deeper understanding of these issues to inform the Patient Safety Education Framework. Parental feedback provides rich insight to help uncover blind spots and improve safety and quality of care.^23^

Key findings related to taking on more child‐ and family‐centred perspectives when defining patient safety, higher safety risk profiles in these children and the power imbalance that exist between parents and staff compounded by staff bias, negative attitudes and diagnostic overshadowing. Communication issues featured highly in a number of themes and how it then relates to developing partnerships with parents, teamwork, care coordination and systems, exposing vulnerabilities in human factors and the health system in identifying and managing risks effectively.

## 6 RETHINKING PATIENT SAFETY

Parents saw safety as going beyond the boundaries of traditional patient safety concepts. For example, the definition of safety is not only the absence of physical harm but includes psychological and emotional harm, feeling safe, a nontraumatic experience for both the child with intellectual disability and the parent. Using the social model in understanding the experiences of the child with an intellectual disability helps health providers become aware that children with intellectual disability and their parents often suffer from barriers inherent in society, for example, bias and negative attitudes, system and environmental structures that disempower and make their plight invisible to the system.^17^ Such feedback is particularly noteworthy and need to be incorporated into the adapted framework.

## 7 SHARED ISSUES WITH IMPLICATIONS FOR ALL PATIENTS

Parents of children with intellectual disability provided a balance of viewpoints highlighting both the difficulties and the triumphs of staff working successfully in collaboration, planning, preparation and addressing system‐related issues. While these issues were found for the intellectual disability population, many of these issues were also shared with the nonintellectual disability paediatric and adult intellectual disability population for example, issues with communication, parent partnerships, information transfer, teamwork, and care coordination^24^, ^25^, ^26^, ^27^, ^28^ and so forth. While improving care for this population is postulated to also have broader benefits to paediatric and adult patients alike, literature assert that children with special needs can have more complex medical and disability needs, requiring a higher level of care coordination and interagency and team collaborations.^29^, ^30^ In addition, what appears to be the most significant are the issues relating to staff attitudes towards caring for a child with intellectual disability and ‘willingness to go the distance’ in terms of providing disability specific adaptations.^12^, ^31^, ^32^, ^33^

### 7.1 How does this add to the Adapted PSEF?

#### 7.1.1 Contributors to risk

Parents of children with intellectual disability raised numerous issues ranging from staff communication and attitudes; lapses in medication management; knowledge gaps in developmental and intellectual disability health, behaviour management, child engagement, admission planning and process implementation, environmental modifications and inclusive practices. It was difficult to ascertain from parents how actual workload issues and stress impacted their experiences of staff, but there were reports of a sense of busyness leading parents to feel that they could not leave their child with intellectual disability to be tended by staff.^20^

#### 7.1.2 Need for training

Communicating with children or patients with an intellectual disability need to be taught to junior staff while they are still learning how to manage doctor–patient/parent relationships and providing safe care. This also mitigates negative habits being carried into their senior years. Knowing how to listen and not make assumptions, learning how to detect distress or change and maintaining good professional conduct were skills parents of children with intellectual disability felt should be instilled.

#### 7.1.3 Addressing unconscious bias and negative attitudes

It has been shown that healthcare staff may harbour negative attitudes towards patients with intellectual disability.^34^ There is significant evidence demonstrating the effects of unconscious bias effects on clinical judgement, one of its unintended outcomes being diagnostic overshadowing.^35^ It is known that staff make assumptions about presenting behaviours due to the intellectual disability and not an indication of symptoms of an organic condition.^6^, ^7^, ^31^ When examining the types of bias known to be associated with diagnostic overshadowing, we found examples of *anchoring bias*, for example, fixed belief despite contrary info, *premature closure*, for example, conclusions made without proper investigation and *implicit bias*, for example, bias from a personal characteristic, for example, age, race, and diagnosis leading to errors being made.^36^ In addition, parents reported that these reactions have led to missed diagnoses, ineffective management, and disrespectful interactions.^2^ Hence, adaptations required in Domain 1 (Communicating Effectively), Domain 2 (Identifying, preventing and managing adverse events and near misses and Domain 4 (Working Safety) need to raise awareness that these biases, conscious or unconscious) exist in the health workforce and provide strategies to discard stereotyping and increase staff desire for greater understanding, compassion and advocacy. In addition, by including strategies and safeguards to assist staff to increase their ability to reflect and scrutinise their clinical decision making to minimise diagnostic overshadowing is also of critical importance (relevant for adaptations in Domain 6 (Continuing Learning). Efforts to address bias through education and exposure to ‘bias literacy’ have reaped benefits in promoting constant self‐evaluation, reflection, and the willingness to change.^35^, ^37^

#### 7.1.4 Ethical responsibility

Ethical conduct requires staff to be sensitive, respectful and responsive to patients, parents or carers/family members, especially during situations when the child with intellectual disability, their parent/carer raise concerns about near misses and safety issues, consent or in shared decision making. Patient values or views should not be dismissed on account of preconceived ideas or biases.^38^ Accurate and honest information needs to be consistently provided and opportunities to help parents feel heard and part of the decision making is important to reduce parental anxiety and contribute to building trust.^39^ Mutual trust and role negotiation is required for the safety and quality of healthcare provision.^39^, ^40^

Health organisations and educational institutions have the ethical duty to ensure that their staff and students are appropriately trained and resourced to carry out safe and good care. Undergraduate and postgraduate programmes have the responsibility of ensuring that intellectual disability health is incorporated into their training programmes.^41^, ^42^ Hence, Domain 5 (Being ethical) has implications not only within the service delivery context but also in the undergraduate, graduate and postgraduate health professional programmes.

#### 7.1.5 Improving communication

Whilst *communicating safely in developmentally appropriate ways* is promoted in the adapted version of the framework (includes competency in the use of communication strategies and resources), there are specific areas that this needs particular emphasis. This includes explaining the risks and benefits of a treatment or procedure, obtaining consent especially in the context of withdrawal of treatment. These sensitive and controversial areas require guidance with clear policy and guidelines and governance oversight to ensure that ethical, sensitive and developmentally appropriate ways have been utilised for optimising outcomes. Providing timely and accurate information then checking back on the child or young person with intellectual disability and their parents or carers/family members is also needed for greater inclusion. These need to be incorporated into Domain 1 (Communicating Effectively) and Domain 2 (Identifying, preventing and managing adverse events and near misses).

#### 7.1.6 Addressing staff wellbeing

Multiple factors influence the way staff think and behave; work stress and burnout also play a part in addition to past experiences and a lack of training resulting in preconceived bias and diagnostic overshadowing.^1^, ^20^ Parents of children with intellectual disability allude to senior staff at times having more negative attitudes than junior staff and attributed this to their prior negative experiences with other parents. However, junior staff are more prone to errors due to their lack of experience. Hence, training and mentorship of staff across seniorities and those who carry clinical and management responsibilities needs to be included in training and education considerations. Organisational support is required to ensure staff are well supported to prevent fatigue, errors and promote empowerment through training and education in intellectual disability health. This pertains to adaptations in Domain 4 (Working Safely) and Domain 6 (Continuing Learning).

#### 7.1.7 Teamwork and care coordination

Team coordination was raised as a critical component of reasonable adjustments as caring for a child with intellectual disability is complex involving many specialities. Good and safe care coordination ensures that ‘everything should happen, does happen at the right time, place, with the right person’. Coordinating outpatient appointments have been reported as a particular challenge and more efforts are needed in liaising with parent/carer/disability support providers to ensure smooth continuity of care. Calling upon the child and young person's disability support worker or therapist to provide information, offer strategies and to be ready to provide support on discharge is often an important step that is overlooked. The appointment of an intellectual disability liaison staff member could be instrumental in improving care coordination especially during points of transition.^43^ These pertain to adaptations in Domain 4 (Working Safely).

#### 7.1.8 Engaging the child reaps multiple benefits for safety

Spending time getting to know a child with an intellectual disability recognises their personhood. It builds trust and enables parents to feel confident about leaving their child in the care of staff. Using one's interpersonal skills to engage with a child assists in making them feel safe and comfortable especially when needing to undergo a procedure or uncomfortable treatments.^44^ The ability to successfully establish rapport and provide child‐centred care brings job satisfaction and reward, moving staff from fear and uncertainty to joy and self‐agency as a paediatric health professional resulting in safer and better care for their patients with intellectual disability.^44^, ^45^ These have implications for training and relevant in Domain 6 (Continuing Learning).

#### 7.1.9 Better integration between child‐ and family‐centred approaches with processes within the system

Parents of children with intellectual disability highlighted their need to be included in decisions concerning their child. This includes clinical discussions, method of child engagement, ensuring IT systems and processes are aligned to the needs of their child, preparation of/for procedures and admissions, environmental modification, customisation of care, transfer of information and team handover. Child‐ and family‐centred approaches ensure they remain at the heart of all clinical interactions including system and process considerations.^40^, ^46^

Lack of links to electronic records and information were identified; ones that lead to near misses or adverse events—also cited in literature raising issues of patient safety for children with intellectual disability.^47^ Improving information linkage and staff access will improve communication and information transfer leading to safer and better care.^48^ This pertains to adaptations in Domain 1 (Communicating Effectively), Domain 4 (Working Safely) and Domain 3 (Using Evidence and Information).

#### 7.1.10 Power Imbalances

Parents of children with intellectual disability did not always feel empowered to escalate issues because of the fear of repercussions in the care of their child. One study showed that even when parents were given a resource Call for Help (C4H), parents still lacked confidence to raise concerns about interprofessional communication.^49^ This disempowerment is a latent issue; one with the potential for an adverse event as it curtails parent's ability to raise the alarm when mistakes occur. Health staff need to understand power dynamics that inhibit authentic staff–parent partnership.^50^ Enabling parents to speak up in escalating care is increasingly being recognised as an important role in serious life‐threatening conditions.^51^

When parents and families are provided with information, seen as partners and given desired opportunities to provide technical care for their child, for example such as changing dressings, tube feeding or suctioning their airways, their anxieties are alleviated giving them a sense of control.^39^ Helping parents feel valued contributes to openness and shared care responsibilities. This allows the development of trusting relationships with the treating team.^39^, ^40^ Findings from several studies indicate that patients who are involved with care decisions and management have better outcomes than those who are not.^3^, ^40^

Inclusion of these key learning points is relevant in Domain 1 (Communicating Effectively), Domain 2 ((Identifying, preventing and managing adverse events and near misses), Domain 4 (Working Safely) and Domain 5 (Being ethical).

#### 7.1.11 Additional topics

Inclusion of topics around medication management specific to children with intellectual disability; access to information on comorbidities; syndromes and rare genetic conditions; use of communication strategies; behaviour management; assessing pain, distress or deterioration; specific equipment and environmental modifications; improving processes in identifying need for adjustments was recommended. We are undertaking a Delphi process with expert stakeholders to look at the need for new subheadings special topics and/or integrate these into existing domains (Supporting Information S1: Data 1).

## 8 LIMITATIONS

The study recruitment was limited only to families and children with intellectual disability attending two tertiary paediatric centres with relatively small numbers. However, data saturation was achieved through cocoding and consensus discussions with the research team. Most of the themes were also found to be congruent with pre‐existing literature in this area. Next steps will be undertaken to triangulate the data with literature reviews specific to the domains of patient safety and compare this with data from staff interviews.

## 9 CONCLUSION

This study identifies the parent experience themes that will inform the development of a Patient Safety Education Framework for children with intellectual disability in the hospital. It highlights the importance of staff reflexivity in addressing negative attitudes and biases towards children with intellectual disability and in planning and providing for their care. Education programmes that focus on competencies such as engaging with a child with an intellectual disability, delivering patient‐ and family‐centred care, addressing systems issues, and providing reasonably adapted care, these would serve as cornerstones for the development of staff competencies of the adapted framework.

## AUTHOR CONTRIBUTIONS

Natalie Ong contributed to the design, conduct, data analysis and writing up of the study. Abbie Lucien contributed to the data analysis and writing up of the study. Janet Long, Janelle Weise, Annette Burgess and Merrilyn Walton contributed to the design of the study, discussion of the results and writing up of the study.

## CONFLICT OF INTEREST STATEMENT

The authors declare no conflict of interest.

## ETHICS STATEMENT

Ethics approval was granted by the Sydney Children's Hospitals Network Human Research Ethics Committee: 2020ETH02240. All participants in this study provided a signed consent form returned to the research team.

## Supporting information

Supporting information.

## Data Availability

The data that support the findings of this study are available from the corresponding author upon reasonable request.
