# Supplementary material for: What do parents think about the quality and safety of care provided by hospitals to children and young people with an intellectual disability? A qualitative study using thematic analysis
Source: Health Expect. 2023 Nov 28;27(1):e13925. doi: 10.1111/hex.13925 (PMC10768875; doi:10.1111/hex.13925)
Supplement: Supplementary file 1 — Supporting information. [file HEX-27-e13925-s001.docx]

| **Australian Patient Safety Education Framework** | **Adapted PSEF for children or young persons with intellectual disability** |  |
| --- | --- | --- |
| **Domain 1: Communicating Effectively** | **Domain 1: Communicating Effectively** | **Key Points included in subdomains** |
| Subdomain | Subdomain |  |
| 1.1 Involving patients and carers as partners in health care | 1.1 Involving the child or young person with intellectual disability, parents, and carers as partners in health care | Use of communication strategies and resources  Working with parents |
| 1.2 Communicating risk | 1.2 Communicating with the child or young person with intellectual disability and their families using developmentally appropriate and adjusted ways |  |
| 1.3 Communicating honestly with patients after an adverse event (open disclosure) | 1.3 Adapting Open disclosure practices |  |
| 1.4 Obtaining consent | 1.4 Adaptations for obtaining valid informed consent |  |
| 1.5 Being culturally respectful and knowledgeable | 1.5 Being inclusive, respectful and responsive in the context of a child or young person with intellectual disability |  |
| **Domain 2: Identifying, preventing, and managing adverse events and near misses** | **Domain 2: Identifying, preventing, and managing adverse events and near misses** |  |
| 2.1 Recognising, reporting, and managing adverse events and near misses | 2.1 Improving practice in recognising, reporting and managing adverse events and near misses for the child or young person with intellectual disability | Identifying deteriorating states, pain or distress |
| 2.2 Managing risk | 2.2 Managing risk in the disability context |  |
| 2.3 Understanding healthcare adverse events and near misses | 2.3 Engaging with the child or young person with intellectual disability, parents/ carers during complaints processes |  |
| 2.4 Managing complaints |  |  |
| **Domain 3: Using evidence and information** | **Domain 3: Using evidence and information** |  |
| 3.1 Employing best available evidence-based practice | 3.1 Employing best available evidence about safety and quality in care for child or young person with intellectual disability | Understanding limited evidence but able to adapt and seek advice |
| 3.2 Using information technology to enhance safety | 3.2 Using information technology to identify and assist staff caring for the child or young person with intellectual disability in identifying the need for reasonable adjustments |  |
| **Domain 4: Working Safely** | **Domain 4: Working Safely** |  |
| 4.1 Being a team player and showing leadership | 4.1 Becoming a local champion and harnessing teamwork in improving care for children and young people with intellectual disability |  |
| 4.2 Understanding human factors | 4.2 Understanding human factors, attitudes, bias, and diagnostic overshadowing |  |
| 4.3 Understanding complex organisations | 4.3 Understanding complex organisations, processes and systems issues impacting care of the child or young person with intellectual disability |  |
| 4.4 Providing continuity of care | 4.4 Providing continuity of care and transition to adult services |  |
| 4.5 Managing fatigue and stress | 4.5 Managing fatigue and stress and its influence on emotional care |  |
| **Domain 5: Being ethical** | **Domain 5: Being ethical** |  |
| 5.1 Maintaining fitness to work and practice | 5.1 Developing skills to provide care for chronic and complex conditions and manage behaviours that challenge |  |
| 5.2 Professional and ethical behaviour | 5.2 Professional and ethical behaviour | Patience, Flexibility, Avoiding diagnostic overshadowing |
| **Domain 6: Continuing Learning** | **Domain 6: Continuing Learning** |  |
| 6.1 Being a workplace learner | 6.1 Being a workplace learner in intellectual disability health |  |
| 6.2 Being a workplace teacher | 6.2 Being a workplace teacher in intellectual disability health |  |
| **Domain 7: Specific Issues** | **Domain 7: Specific Issues** |  |
| 7.1 Preventing the wrong site, wrong procedure, and wrong patient treatment | 7.1 Preventing or reducing errors in diagnosing and managing health conditions and child or young person with intellectual disability | This includes the need for staff to be able to:   1. Identify deteriorating states 2. Assess pain or distress 3. Know how to get advice or info about rare genetic conditions 4. Transfer of care – include disability info |
| 7.2 Medicating safely | 7.2 Medication safely in the context of child or young person with intellectual disability | Polypharmacy, Use of meds for managing behaviours that challenge |
|  | 7.3 Supporting the child or young person with intellectual disability and parents with vulnerabilities |  |
|  | 7.4 Child protection considerations for the child or young person with intellectual disability | Not only risk to child but risk to parents from abusive child |
|  | 7.5 Understanding interplay of co-morbidities eg. autism, ADHD, anxiety |  |
|  | Other? |  |

Supplementary Data 1. APSEF old and new domains.docx
